# Cognitive Neuroscience Meets the Community of Knowledge

**DOI:** 10.3389/fnsys.2021.675127

**Published:** 2021-10-21

**Authors:** Steven A. Sloman, Richard Patterson, Aron K. Barbey

**Affiliations:** ^1^Department of Cognitive, Linguistic, and Psychological Sciences, Brown University, Providence, RI, United States; ^2^Department of Philosophy, Emory University, Atlanta, GA, United States; ^3^Department of Psychology, University of Illinois at Urbana-Champaign, Champaign, IL, United States; ^4^Department of Bioengineering, University of Illinois at Urbana-Champaign, Champaign, IL, United States; ^5^Neuroscience Program, University of Illinois at Urbana-Champaign, Champaign, IL, United States; ^6^Beckman Institute for Advanced Science and Technology, University of Illinois at Urbana-Champaign, Champaign, IL, United States

**Keywords:** community of knowledge, cognitive neuroscience, thinking, collective cognition, social neuroscience

## Abstract

Cognitive neuroscience seeks to discover the biological foundations of the human mind. One goal is to explain how mental operations are generated by the information processing architecture of the human brain. Our aim is to assess whether this is a well-defined objective. Our contention will be that it is not because the information processing of any given individual is not contained entirely within that individual’s brain. Rather, it typically includes components situated in the heads of others, in addition to being distributed across parts of the individual’s body and physical environment. Our focus here will be on cognition distributed across individuals, or on what we call the “community of knowledge,” the challenge that poses for reduction of cognition to neurobiology and the contribution of cognitive neuroscience to the study of communal processes.

## The Individual Brain and Collective Mind

A central aim of cognitive neuroscience is to explain how people think, elucidating the representations and processes that allow humans to judge, reason, remember, and decide (Barbey et al., 2021). To achieve this goal, cognitive neuroscientific theories have as a rule made certain fundamental assumptions:

(a) Knowledge is represented in the brain.
(b) Knowledge is represented by the individual.
(c) Knowledge is transferred between individuals.

where “knowledge” is understood broadly—as it usually is in behavioral science—as people’s attempts to represent their world, including both observable and latent objects and processes, in ways that support memory, understanding, reasoning, and decision making. It includes beliefs that are more or less justified, and that might correspond to factual truth or not. Evidence to suggest that knowledge is represented in the brain [assumption (a)] may reflect: (1) correlations with neural activity (e.g., spike trains generated by neurons in V1 correlate with the presence and location of edges in the visual environment), (2) causal effects of knowledge on the operation of neural systems (e.g., spike trains generated by neurons in V1 are used by downstream areas for further processing), and/or (3) neural computations applied to manipulate and process knowledge. Although assumption (a) is typical of theories in the psychological and brain sciences (for reviews, see Gazzaniga et al., 2019; Barbey et al., 2021), it is not universal. Proponents of embodied cognition see knowledge as distributed across the brain, the body, and artifacts used to process information (e.g., Barsalou, 2008) and proponents of cultural psychology sometimes see knowledge as embedded in cultural practices (Duque et al., 2010; Holmes, 2020). But assumptions (b) and (c) are widely shared by disciplines that focus on cognition (for a review, see Boone and Piccinini, 2016). The idea is that what really counts as cognition is mediated by individual processes of reasoning and decision making; that cognitive processing is distinct from interactions with books, the internet, other people, and so on. Moreover, other people are obviously sources of information, but their value for an individual is in the information they transfer. The goal of this manuscript is to question the generality of these assumptions, spell out some of the resulting limitations of the cognitive neuroscience approach, and try to suggest some more constructive directions for the field. Our contention will be that the information processing of any given individual is not contained entirely within that individual’s brain (or even their bodies or physical environments). Rather, it typically includes components situated in the heads of others, and that the transfer of information is more the exception than the rule.

Assumption (a) as usually understood implies (b). If knowledge is represented in the brain, then it is represented by individuals. Thus standard neuroimaging methods assess brain activity and task performance within the individual (for a review of fMRI methods, see Bandettini, 2012). According to this view, the neural foundations of the human mind can be discovered by studying the individual brain and identifying common patterns of brain activity across individuals. Thus, by averaging data from multiple subjects, cognitive neuroscience seeks to derive general principles of brain function and thereby reveal the mechanisms that drive human cognition. This approach lies at the heart of modern research in cognitive neuroscience, reflecting a disciplinary aim to generalize beyond the individual to characterize fundamental properties of the human mind using widely held methodological conventions, such as averaging data from multiple subjects, to infer general principles of brain function (Gazzaniga et al., 2019).

Although assumption (a) implies (b), the converse does not also hold. If knowledge is represented by the individual, it need not be represented exclusively within the brain. More importantly, as we will argue, an individual’s knowledge not only arises in large part from communal interactions, but also depends on cognitive states of other members of the community. This places limits on the utility of studying individual brains to infer general principles of the collective mind. Our conclusion is decidedly not that cognitive neuroscience makes no contribution to the study of cognition. It is that cognitive neuroscience does not provide a sufficient basis to model cognition. Social neuroscience is an emerging field that addresses part of the problem, as it takes as a central tenet that “brains are not solitary information processing devices” (Cacioppo and Decety, 2011). Nevertheless, the discussions we are aware of within the field of cognitive neuroscience still abide by assumptions (b) and (c).

## The Community of Knowledge and the Limits of the Individual

We start with assumption (b). Years of research in psychology, cognitive science, philosophy, and anthropology have shown that human cognition is a collective enterprise and is therefore not to be found within a single individual. Human cognition is an emergent property that reflects communal knowledge and representations that are distributed within a community (Hutchins, 1995; Clark and Chalmers, 1998; Wilson and Keil, 1998; Henrich, 2015; Mercier and Sperber, 2017; Sloman and Fernbach, 2017). By “emergent” property we mean nothing elusive or mysterious, but simply certain well-documented properties of groups that would not exist in the absence of relevant properties of individuals, but are not properties of any individual member of the group, or any aggregation of properties of some or all members of the group.

Accumulating evidence indicates that memory, reasoning, decision-making, and other higher-level functions take place across people. The evidence that mental processing is engaged by a community of knowledge is multifaceted (for a review, see Rabb et al., 2019). The claim that the mind is a social entity is an extension of the extended mind hypothesis (Clark and Chalmers, 1998): Cognition extends into the physical world and the brains of others. The point is not that other people know things that I do not; the point is that my knowledge often *depends* on what others know even in the absence of any knowledge transfer from them to me. I might say, “I know how to get to Montreal,” when what I really mean is that I know how to get to the airport and the team piloting the aircraft can get from the airport to Montreal. Similarly, one might say that “what makes a car go” is the motor: that’s why it’s called a “motor,” after all. But while a full account will include the engine as a key contributor, the propulsion system is distributed over the engine, drive shaft, the human who turns the key, fuel, a roadway, and more. Changing the boundaries of what has traditionally been considered cognitive processing in an analogous way – from individual brains to interacting communities – perhaps raises questions of who should get credit and who should take responsibility for the effects of an individual’s action, but it is nevertheless an accurate description of the mechanisms humans use to process information. Furthermore, as the boundaries for what counts as cognitive processing shift, the operational target for studying the human mind moves beyond the scope of methods that examine performance through the lens of the individual.

Philosophers analyzing natural language illustrate how cognitive processes are extended into the world. The classic analysis is by Putnam (1975), who points out that we often use words whose reference (or denotation or extension) and therefore, according to Putnam, their meaning, is determined by factors outside one’s brain or mind (i.e., externalism). One could see Humpty Dumpty as an extreme and defiant internalist: “When I use a word, it means precisely what I want it to mean, no more and no less” (Carroll, 1872). Putnam’s argument is the subject of vigorous and sophisticated but not entirely conclusive debate (Goldberg, 2016; see also Burge, 1979). Nonetheless it is now widely agreed that some form of externalism is at least a necessary part of an explanation of how our everyday terms have their referents (or denotations) and meanings.

The philosopher whom one might call the Godfather of Externalism, Wittgenstein (1973), preferred to draw attention to what he saw as linguistic facts that had been overlooked, above all, that the meaning of words depends on (or is even identical to) their use. Although that bald statement is highly controversial, what matters from our point of view is that the meaning of a word and its correct use depend on collective knowledge that extends beyond the individual, reflecting a social context (Boroditsky and Gaby, 2010). Thus, for a community of knowledge to support meaning and communication, there must be sufficient stability of common usage even as usage typically changes over time. The same holds for sentence meanings, as in, “Zirconium comes after Yttrium in the Periodic Table.” The speaker may have long ago forgotten—or never even knew—what exactly Zirconium is and why one thing comes after another in the Periodic Table. Nonetheless the statement has a meaning that has been fixed by the appropriate members of the scientific community, and propagated more-or-less successfully to generations of students. The speaker’s statement is true and has that communally established meaning, no matter how confused the speaker may be. Some might distinguish the speaker’s meaning from the correct, communally-ordained meaning. That is important in some contexts (e.g., in teaching and in evaluating students), but the point here is that the sentence has a precise meaning established by chemical science, even if that is not precisely what is in the speaker’s head, but only in the heads of others.

The same holds of theories. The statement “According to modern chemistry there are more than a hundred elements” is true regardless of how well or poorly the speaker might understand modern chemistry. It is true because “modern chemistry” means the chemical theories agreed upon by socially recognized experts. This holds even if the relevant theories are no longer in the speaker’s head, and even if the speaker never understood the theories.

These remarks on social meaning converge with recent work in the emerging discipline of “social epistemology” (Goldman, 1999), the study of knowledge as a social entity. We will speak of “knowledge” in an everyday sense, without entering into the labyrinthine and ultimately inconclusive attempts at definition offered by philosophers from the time of Plato, including what “really constitutes” social knowledge. What matters here is that research within social epistemology demonstrates that successful transmission of knowledge clearly does occur and depends on three general conditions (Goldberg, 2016): (i) social norms of assertion; (ii) reliable means of comprehending what is said (which depend on social norms of meaning and usage); and (iii) a reliable way of telling a reliable source of knowledge from an unreliable one. For reasons we elaborate below, we believe that the role of society in epistemology is not only to transmit knowledge from one individual to another, but to retain knowledge even when it is not transmitted.

Sloman and Fernbach (2017) extended the externalist project well beyond a concern with the meanings of words, to large swathes of conceptual knowledge. Outside their narrow areas of expertise, individuals are relatively ignorant (Zaller, 1992; Dunning, 2011). In any given domain, they know much less than there is to know, but nonetheless do know certain things that others understand more fully. The extent to which we rely on others in this way is often obscured by the fact that people tend to overestimate how much they know about how things work (Rozenblit and Keil, 2002; Lawson, 2006; Fernbach et al., 2013; Vitriol and Marsh, 2018). They overestimate their ability to reason causally (Sloman and Fernbach, 2017). They also overestimate what they know about concept meanings (Kominsky and Keil, 2014) and their ability to justify an argument (Fisher and Keil, 2014) and claim to have knowledge of events and concepts that are fabricated (Paulhus et al., 2003).

The best explanation for our tendency to overestimate how much we know is that we confuse what others know for what we know (Wilson and Keil, 1998). Others know how things work, and we sometimes fail to distinguish their knowledge from our own. The idea is the converse of the curse of knowledge (Nickerson, 1999). In that case, people tend to believe that others know what they themselves know (this is part of what makes teaching hard). In both cases, people are failing to note the boundary among individuals. Circumstances can produce a rude awakening if things go wrong and we suddenly need to understand how to fix them, or if we are otherwise challenged to produce a full explanation either in a real world situation or by a psychologist.

Nonetheless, as Goldman (1999) observes, even a shallow understanding of a concept, idea, or statement can give us valuable practical information. Fortunately, we can know and make use of a good many truths without ourselves possessing the wherewithal to prove them, so long as our limited understanding is properly anchored elsewhere. We develop multiple examples below. Meanwhile, from a very broad perspective, we note that the conceptual web is tangled and immense, containing far more than a mere mortal could store and make sense of Sloman and Fernbach (2017). Thus we are by nature creatures that rely heavily on others to have full understandings of word meanings (“semantic deference” in the philosophical literature) and a more full and secure grasp of ideas, statements, or theories than our own incomplete grasp reflected in our shallow understanding. This dovetails not only with experimental results (Rozenblit and Keil, 2002; Fernbach et al., 2013; Kominsky and Keil, 2014; Sloman and Rabb, 2016), but also with recent anthropological work on culture-gene coevolution showing that cultural accumulation exerted selective pressure for genetic evolution of our abilities to identify and access reliable sources of information and expertise (e.g., Richerson et al., 2010; Henrich, 2015).

At a social level, the fact that knowledge is communal also has a political dimension. As societies develop, group policy and decision-making will depend on the aggregation, coordination, and codification of various sorts of knowledge distributed across many individuals (e.g., experts in the production, storage, distribution, and preparation of food). There is lively debate among political theorists about whether command and control societies, democracies, or something else can best fulfill the needs and aspirations of its members (Anderson, 2006; Ober, 2008). Is decision-making best served by cloistered experts or through information gathered from non-experts as well? Non-experts presumably have greater access to details of local situations, but attempts to utilize widely distributed knowledge poses greater problems of aggregation and coordination. As Hayek (1945) remarked, the aggregation and deployment of widely distributed information is a central issue for theories of government. However, our interest here is not the relative merits of different forms of government. We mention these issues only to illustrate the far-reaching and pervasive importance of information processing in social networks and by implication the need for a political level of explanation in the understanding of a community of knowledge.

## Social Knowledge Without Social Transmission: Outsourcing

Work on collective cognition points to several ways that individual cognition depends on others (Hemmatian and Sloman, 2018). One is collaboration: Problem-solving, decision-making, memory, and other cognitive processes involve the joint activity of more than one person, and in many contexts mutual awareness of a joint intention to perform some task. Work on collaboration has focused on team dynamics (Pentland, 2012) and group intelligence (Woolley et al., 2010). A second form of cognitive dependence on others, and the one that grounds our argument, is outsourcing: The knowledge people use often sits (or sat) in the head of someone else, someone not necessarily present (or even alive). Frequently, outsourcing requires that we have *access* to outsourced knowledge when the need arises. But often merely knowing we have access is sufficient for practical purposes (e.g., we go to Tahiti assuming we’ll find what we need to enjoy ourselves when we’re there). On occasion we do access the information, and this requires some type of social transmission. Such transmission comes in the form of social learning of a skill, practice, norm, or theory on the one hand, or in the form of more episodic or *ad hoc* accessing of information for limited, perhaps one-time, use (Barsalou, 1983). A prime example of the former would be an apprentice learning a trade from a master; of the latter, “googling” to find out who won the 1912 World Series. The transmission of information around a social network is a key determinant of human behavior (Christakis and Fowler, 2009).

A key requirement in using information that is sitting in someone else’s head is the possession of what we will call epistemic pointers (“epistemic” meaning having to do with knowledge): the conscious or implicit awareness of where some needed information can be found. Sometimes we can envision many potential pathways to an information source, whether direct or indirect, and sometimes very few. Thus we may envision many potential information sources for how to get to Rome (travel agents, friends who have been there), and various pathways by which we might access a given source (e.g., find the phone number of a friend who said she had a good travel agent) but fewer pathways to find out how to get to the rock shaped like an elephant that someone mentioned in passing. Our representations of pointers, to a source or to a step on a pathway to a source, can be partial and vague, providing little or no practical guidance (“some physics Professor knows it”), or full and precise (“it’s in Einstein’s manuscript on the special theory of relativity”). If we are completely clueless, we can be said to lack pointers and pathways, and simply have a placeholder for information. The evidence of human ignorance that we review below leads us to suspect that the vast majority of the knowledge that we have access to and use is in the form of placeholders.

## Setting the Stage: Collaboration

The centrality of collaboration for human activity derives from the fact that humans are unique in the cognitive tools they have for collaboration. Tomasello and Carpenter (2007) make the case that no other animal can share intentionality in the way that humans can in the sense of establishing common ground to jointly pursue a common goal, and a large body of work describes the unique tools humans have to model the thoughts and feelings, including intentions and motivations, of those around them (e.g., Baron-Cohen, 1991).

The role of collaboration in specifically cognitive performance has been most fully studied in memory. Wegner et al. (1991) report some of the early work showing that groups, especially married couples, distribute storage demands according to relative expertise. They call these “transactive memory systems.” Theiner (2013) argues that transactive memory systems reflect emergent group-level memories, providing evidence that: (i) members of a transactive memory system are not interchangeable (because each member makes unique contributions to the group); (ii) if members are removed from the group, the system will no longer function (omitting essential components of the group-level memory); (iii) the disassembly and reassembly of the group may impair its function (for example, when members of the group no longer understand the distribution of knowledge within the system and what information they are responsible for knowing); and (iv) cooperative and inhibitory actions among members are critical (given the interactive and emergent nature of transactive memories) (for a review, see Meade et al., 2018). Wilson (2005) claims that these properties of a transactive memory system have important political consequences as they affect the commemoration and memorialization of politically relevant events and culturally important origin stories that shape nationalism and attitudes toward human rights and other issues. Memory systems play a critical role in communities.

Further evidence for the importance of collaboration in thought comes from naturalistic studies of group behavior. The seminal work was conducted by Hutchins (1995). He offered a classic description of navigating a Navy ship to harbor, a complex and risky task. The process involves multiple people contributing to a dynamic representation of the ship’s changing location with reference to a target channel while looking out for changing currents and other vessels. Various forms of representation are used, all feeding into performance of a distributed task with a common goal. Sometimes the common goal is known only by leadership (in the case of a secret mission, say). Nevertheless, successful collaboration involves individuals pursuing their goals so as to contribute to the common goal. Many of the tasks we perform everyday have this collaborative nature, from shopping to crossing the street. If a car is coming as we cross, we trust that the driver won’t accelerate into us, and the more assertive street crossers among us expect them to slow down in order to obtain the common goal of traffic flow without harm to anyone. Banks and Millward (2000) discuss the nature of distributed representation and review data showing that distributing the components of a task across a group so that each member is a resident expert can lead to better performance than giving everyone the same shared information. Hutchin’s nautical example illustrates this, insofar as some essential jobs require multiple types of expertise. Other jobs might not require this, so that crew members may substitute for one another, because all of them have the same basic information or skill level needed for the job. Often in real life there will be a mix, so that the task occupies an intermediate position relative to Banks and Millward’s two types of group (i.e., diverse local experts versus all group members having the same knowledge). Work on collective intelligence also provides a good example of emergent group properties, illustrating how collective problem-solving relies more on collaboration and social interconnectedness than on having individual experts on the team (Woolley et al., 2010).

## Collaboration and Neuroscience: The Case of Neural Coupling

Research in cognitive neuroscience has not ignored these trends in the study of cognition. An emerging area of research investigates the communal nature of brain networks, examining how the coupling of brain-to-brain networks enables pairs of individuals or larger groups to interact (Montague et al., 2002; Schilbach et al., 2013; Hasson and Frith, 2016). These studies deploy a generalization of neuroimaging methods, applying techniques that were once used to assess intra-brain connectivity (i.e., within the individual) to examine inter-subject connectivity (i.e., between different subjects; Simony et al., 2016). This can be achieved through experiments in which brain activity within multiple participants is simultaneously examined (i.e., “hyperscanning;” Montague et al., 2002) or analyzed *post hoc* (Babiloni and Astolfi, 2014). Such approaches have been applied to assess brain-to-brain communication dynamics underlying natural language (e.g., Schmalzle et al., 2015). Recently, researchers have placed two people face-to-face in a single scanner to examine, for example, the neural mechanisms underlying social interaction (e.g., when people make eye contact; for a review, see Servick, 2020). The situation – very noisy and now also very crowded – does not score high on ecological validity. Also, it is hard to see how one could scale this approach up to study larger groups (big scanners, little participants?). Nonetheless this is a reasonable place to start, and here, as with hyperscanning and retrospective analysis of neuroimaging data, one might well secure suggestive results. So although the examination of brain-to-brain networks is rare in cognitive neuroscience, with only a handful of studies conducted to date (for a review, see Hasson and Frith, 2016), this approach represents a promising framework for extending cognitive neuroscience beyond the study of individuals to an investigation of dyads, groups, and perhaps one day to larger communities.

This approach has set the stage for research on the neural foundations of communal knowledge, investigating how cognitive and neural representations are distributed within the community and how information propagates through social networks, for example, based on their composition, structure, and dynamics (for a review, see Falk and Bassett, 2017; for a discussion of hyperscanning methods, see Novembre and Iannetti, 2020; Moreau and Dumas, 2021). Evidence from this literature indicates that the strength of the coupling between the neural representation of communication partners is associated with communication success (i.e., successful comprehension of the transmitted signal; Stephens et al., 2010; Silbert et al., 2014; Hasson and Frith, 2016). For example, the degree of brain-to-brain synchrony within networks associated with learning and memory (e.g., the default mode network) predicts successful comprehension and memory of a story told among communication partners (Stephens et al., 2010). Indeed, evidence indicates that people who are closely related within their social network (i.e., individuals with a social distance of one) demonstrate more similar brain responses to a variety of stimuli (e.g., movie clips) relative to individuals who share only distant relations (Parkinson et al., 2017). Research further suggests that the efficiency of inter-subject brain connectivity increases with the level of interaction between subjects, providing evidence that strong social ties predict the efficiency of brain-to-brain network coupling (Toppi et al., 2015; for a discussion of the timescale of social dynamics, see Flack, 2012).

## The Main Event: Outsourcing

A community of knowledge involves more than coupling. We do collaborate, and we engage in joint actions involving shared attention, but we also make use of others without coupling: We outsource to knowledge housed in our culture, beyond the small groups we collaborate with. In the best cases, we outsource to experts. A great many people know that the earth revolves around the sun, but only a much smaller number know how to show that. Both sorts of people are part of a typical community of knowledge, and both are, by community standards, said to know that the earth revolves around the sun. This holds even though the non-expert does not know who the experts are, does not remember how she came to have that knowledge, and does not know what observations and reasoning show that our solar system is heliocentric.

Outsourcing in some circumstances can make us vulnerable to a lack of valuable knowledge. Henrich (2015) describes how an epidemic that killed off many older and more knowledgeable members of the Polar Inuit tribe resulted in the tribe losing access to much of its technology: Weapons, architectural features of their snow homes, and transportation (e.g., a particular type of kayak). Knowledge about how to build and use these tools resided in the heads of those lost members. Without them, the remaining members of the tribe were unable to figure out how to build such tools, and were forced to resort to less effective means of hunting, staying warm, and traveling. The issue here is not collaboration. Tool users were not cognitively coupling with the tool providers. Rather, they were accessing and making use of the latter’s knowledge without acquiring it, in this case outsourcing both the expertise and the production of vital artifacts. Assumptions that individuals had been able to rely on (i.e., that they would have access to a tool for obtaining food) no longer held. The problem was that the younger members of the tribe had outsourced their knowledge to others who were no longer available. Anthropologists have documented numerous cases of loss of technology through death of the possessors of a society’s specialized knowledge, or through isolation from formerly available knowledge sources (e.g., Henrich and Henrich, 2007). By the same token, a community can add new expertise by admitting (or forcibly adding) new members with special skills (e.g., Weatherford, 2005).

Sometimes we are aware that we are outsourcing, for instance when we explicitly decide to let someone else do our cognitive work for us (as one lets an accountant file one’s taxes). In such cases, we explicitly build a pointer, a mental representation that indicates the repository of knowledge we do not ourselves fully possess and that anchors the shallow or incomplete knowledge we do possess. We have a pointer to an accountant or tax lawyer (whether to a specific person or just to a “tax preparer to be determined”), just in case we are audited.

But often we outsource without full awareness, acting as if we have filled gaps in our knowledge even though no information has been transferred. Our use of words is often licensed by knowledge only others have, our explanations often appeal to causal models that sit in the heads of scientists and engineers, and our political beliefs and values are inherited from our spiritual and political communities. More generally, people’s sense of understanding, reasoning, decision-making, and use of words and concepts are often outsourced to others, and often we do not know whom we are outsourcing to, or even that we are doing it. For instance, when we say “*they* landed on the moon,” most of us have little idea who *they* refers to, and often lack conscious awareness that we don’t know who *they* were. Or we say, “We know that Pluto is not strictly speaking a planet.” We know that much on reliable grounds. What little we know is anchored by the possibility of transmission (direct or perhaps very indirect) from communal experts; specifically, the scientists who set the criteria for planethood, and who know whether Pluto qualifies and on the basis of what evidence. Again, it is highly advantageous to be able to outsource – and in fact necessary – since we can’t all master full knowledge of all the crafts, skills, theoretical knowledge, and up-to-date-details of local situations that we need or might need to navigate our environment.

Moreover, people believe they understand the basics of helicopters, toilets, and ballpoint pens even when they do not (Rozenblit and Keil, 2002). Fortunately, others do. In addition, the knowledge that others do increases our sense of understanding not only of artifacts, but of scientific phenomena and political policies (Sloman and Rabb, 2016; Rabb et al., 2019). In fact, just having access to the Internet also increases our sense of understanding even when we are unable to use it (Fisher et al., 2015). These findings cannot be attributed to memory failures because, in the vast majority of cases, the relevant mechanisms were never understood. And the studies include control conditions to rule out alternative explanations based on self-presentation effects and task demands. What they show is that mere access to information increases our sense of understanding. This suggests our sense of understanding reflects our roles as members of a community of knowledge, and suggests that we maintain pointers to or placeholders for information that others retain. The fact that access causes us to attribute greater understanding to ourselves implies that our sense of understanding is inflated. This in turn implies that we fail to distinguish those pointers or placeholders from actual possession of information; we don’t know that we do not really know how artifacts like toilets work, but the awareness that others do leads us to think we ourselves do, at least until we are challenged or we land in a situation demanding genuine expertise (Call the plumber now!).

More evidence for this kind of implicit outsourcing comes from work on what makes an explanation satisfying. People find explanations of value even if they provide no information, as long as the explanations use words that are entrenched in a community. For example, Hemmatian and Sloman (2018) gave subjects a label for a phenomenon (e.g., “Carimaeric”) and told them that the label referred to instances with a specific defining feature (e.g., stars whose size and brightness varied over time). Then the label was used as an explanation for the defining property (someone asked why a particular star’s size and brightness varied over time and was told that it’s because the star is Carimaeric). Subjects were asked to what extent the explanation answered the question. They answered more positively if the label was entrenched within a community than if it was not. Similar findings have been obtained using mental health terms, even among mental health professionals (Hemmatian et al., 2019). In these cases, there is no coupling between the unidentified community members who use the explanation and the agent. There is merely the heuristic that the fact that others know increases my sense of understanding. This heuristic is so powerful that it operates even when others’ knowledge has no informational content.

Some of the clearest evidence for this heuristic comes from the political domain. We often take strong stances on issues that we are ignorant about. These authors believe strongly in anthropogenic climate change despite being relatively ignorant of both the full range of evidence and the mechanism for it. We rely on those scientists who study such things. Political issues tend to be complex and we need to rely on others, at least in part, to form and justify our opinions. In a representative democracy, for instance, we try to be informed on key issues, but rely on specialized committees to investigate matters more thoroughly. For better or for worse, individual support for policies, positions, and leaders comes largely from partisan cues rather than non-partisan weighing of evidence (Cohen, 2003; Hawkins and Nosek, 2012; Anduiza et al., 2013; Han and Federico, 2017; Van Boven et al., 2018). A growing body of evidence indicates that partisan cues determine how we understand events (Jacobson, 2010; Frenda et al., 2013; but see Bullock et al., 2015) and even whether we take steps to protect ourselves from infectious disease (Geana et al., 2021)^1^. Marks et al. (2019) show that people use partisan cues to decide whose advice to follow in a competitive game even when they have objective evidence about who the better players are. When evaluating data, we are often more concerned with being perceived as good community citizens by acceding to our community’s mores than we are with making accurate judgments (Kahan et al., 2011). Such a bias has a rationale if it maintains community membership, and membership is deemed more important than being correct.

Outsourcing knowledge, including the choice of whom to outsource to, is a risky affair. One must estimate what the source does and does not know, their ability to transmit information, and whether their interests align with yours. One must determine how much to trust potential sources of information. Outsourcing, whether influenced by partisan bias or not, is a direct consequence of the human need and tendency to construct pointers to knowledge that other people store.

The basic features of how a community holds knowledge—relative ignorance associated with epistemic pointers to expertise—apply to both social information and disinformation, to well-grounded knowledge, as well as fervently held nonsense perpetrated by unreliable sources. Community norms about what counts as knowledge, and as a reliable pathway of knowledge transmission, may vary greatly: One subculture will require, for some subject matters, scientific expertise on the part of an ultimate source, along with reliable paths of transmission of scientific knowledge, paths often institutionalized, as with schools or trade unions and their certifications. Another subculture will consider God the ultimate source of understanding in important areas, and divine revelation, or the word of officially ordained spokespersons, as appropriate paths of dissemination.

Thus the role of our social networks goes beyond actively sharing information. We use them to represent and process information, such that the network itself serves as an external processor and storage site. We trust others to maintain accurate statistics, to distil news, to total our grocery bill, help us fill out our tax forms, and to tell us what position to take on complex policy. In all such tasks, representation and processing of essential information does not in general occur in individual brains. They do not occur in individual brains even if we allow that those brains are coupled within a social network. Representation and processing occur over a larger portion of an encompassing network, and potentially over the entire network, branching out to include our sources, our sources’ sources, and any intermediaries such as books, the internet, or other people, along the paths of transmission.

## Outsourcing in Cognitive Neuroscience: Constructing Epistemic Pointers

To explain phenomena associated with outsourcing, we cannot appeal to coupling, because coupling requires specification of who is coupling with whom. To explain outsourcing, cognitive neuroscientists must appeal to a different theoretical construct: Neural pointers or placeholders, representations in the brain that act as pointers to knowledge held elsewhere. The work in cognitive neuroscience that most directly addresses the mechanisms of outsourcing concerns how the representation of knowledge relates to affiliation, on whom we trust to retain reliable knowledge. Putting aside the role of trust in institutions, social neuroscience research examining trust in more personal contexts indicates that trust and cooperation are mediated by a network of brain regions that support core social skills, such as the capacity to infer and reason about the mental states of others (for reviews, see Adolphs, 2009; Rilling and Sanfey, 2011). This work provides the basis for future research investigating how the neurobiology of trust contributes to the representation and use of outsourcing in collective cognition. To do so, however, the field will need to move beyond the use of “isolation paradigms” in which subjects observe others whom they might or might not then trust (Becchio et al., 2010). In such cases, subjects neither participate in direct social interaction with potential objects of trust nor outsource their own reasoning to others (Schilbach et al., 2013). Such observation is seldom the sole basis of epistemic pointers, and often is not involved at all. Instead, pointers typically depend on cues that reflect how third parties or the community as a whole regard a potential source. This can involve informal gossip or more institutionalized “rating systems” and reviews, where the latter will bring us back to social institutions. So there is a vast arena, virtually unexplored by social neuroscience, starting with the origin and nature of the neural mechanisms that serve as pointers to communal knowledge.

## The Irreducibility of the Community of Knowledge

The implication of our discussion is that many activities that seem solitary—like writing a scientific paper—require a cultural community as well as the physical world now including the Internet (to ground language, to support claims, to provide inspiration and an audience, etc.). Does this mean there is no solely neurobiological representation for performing such tasks? Perhaps neurobiological reduction can be accomplished by giving up on the idea of reduction to a single brain, and instead appeal to reduction to a network of brains (Falk and Bassett, 2017). Perhaps a broader view of cognitive neuroscience as the study of information processing in a social network of neural networks can overcome the challenge posed for cognitive neuroscience by the community of knowledge. Can networks of individuals processing together be reduced to networks of brains interconnected by some common resource, perhaps some form of neural synchrony?

We believe the answer is “no.” For one thing, the relevant social network is frequently changing, as is membership in groups addressing different problems (for climate change, it involves climate scientists but for predicting football scores, it involves football fans). So there are no fixed neurobiological media to appeal to. This might seem to be irrelevant, as the goal of cognitive neuroscience is not to reduce cognition to a group of specific brains. Rather, one studies specific brains in order to find general patterns of activity that occur in different brains. But this is precisely the problem; namely, the general pattern may not capture specific properties exhibited by the individual. Generalization from the group to the individual depends on equivalence of the mean and variance at each level; an equivalence that has increasingly been called into question (Fisher et al., 2018). The same problem will almost certainly arise with generalizations about multiple groups’ performance of a given task. Indeed the problem may be much worse, as changing group membership may introduce even greater variation across groups of the patterns of interaction that produce a group’s performance.

Changes in membership will not just mean changes in the attributes and resources the members bring to the group, but also – and more strikingly – potentially very large differences in the way members interact, even if they happen to produce the same result (e.g., if they forecast the same football score as another group whose members interacted in their own, different way in arriving at that prediction). Studies of group dynamics and organizational behavior recognize that many factors affect the efficiency and result of group collaboration: the relative dominance of discussion by some particular member(s), the timidity of others, the motivations of members, the level of experience and expertise of the members, the level of relevant knowledge about the particular teams involved, the stakes involved in making a good prediction, time limitations, the degree of synergy among team members, size of the group, form of discussion used (Hirst and Manier, 1996; Cuc et al., 2006), demographic makeup of the members, and so on. Different fans, or even the same fans on different occasions, can arrive at the same score forecasts for the same game by an unlimited number of patterns of interaction. This not only produces the problem of multiple realization (of a type of group performance on a given task) on a grand scale, but indicates that there will be no tolerably definite and generalizable pattern of group dynamics that applies to particular groups addressing the same given task. Hence there is no one general pattern, or even manageable number of patterns, to be reduced to neuroscience.

On a more positive note, research in group dynamics and organizational behavior has, as just noted, identified numerous factors that enter into group performance. So cognitive neuroscience (social and individual) can, by drawing on that research, investigate the neural underpinnings of types of factors such as trust, mind-reading capacities, and many others that drive different forms of group interaction, and this will be essential for an account of group cognition if such an account is ever to be had. But that is a far cry from reducing group behavior to any variety of neuroscience.

## Group Intelligence and Inventiveness

Anthropological and psychological research, in the lab and in the field, strongly reinforces the point: group intelligence and group inventiveness are not just the properties of an individual (such as the smartest or most inventive member of the group), or an average of the members’ properties, or an aggregate of the members’ individual cognitive properties (Woolley et al., 2010). They are sometimes quite surprising properties that emerge from interactions among members of the group, in some cases as a matter of learning, sometimes just from a repeated exchange of ideas, sometimes from a group of initially equal members, sometimes from a group with one or two initial stand outs. The effect of group interaction can be positive or negative depending on the motivations, personal traits, group camaraderie and various situational constraints (e.g., time limitations, availability of paper and pencil, food, and rest).

The moral is that examination of the brains of group members will not reveal or predict precisely how the group as a whole will perform, nor through what complex pattern of interaction or mechanisms it arrived at a given result. Even in a relatively small group there will be an enormous number of interactions that might produce any given result, and that number will increase exponentially with any increase in group size, not to mention the introduction of other potentially influential factors.

Thus there is no way to identify any particular neurobiological pattern (or manageably small number of patterns) across brains as *the* way(s) in which groups produce new knowledge, or even the way the same group functions on different occasions or with regard to different sorts of cognitive tasks. Put another way, even if we could find out through observation, self-report, or fMRI conducted in everyone, that specific members of a given group engaged in certain specific types of interaction with other specific members, and we were able to reduce that to neurobiological terms, we would not be able to say more than that this is one of innumerable ways a particular group result might be realized in a particular social and physical context. An open-ended list of possible realizations at the psychological or behavioral level does not support a reduction of this bit of psychological description to cognitive neuroscience even if it tells us a lot about what goes into that performance. Note once again that we need functional descriptions, which will themselves be complex and predictive of behavior in only a limited way. Functional descriptions will, as with individual psychology and neuroscience, provide essential guidance and support for social neuroscience, and potentially draw on insights from neuroscience.

## Justification and Communal Norms

We saw earlier that within a community of knowledge most of what we know is anchored in the heads of people doing scientific, technical, and other sorts of intellectual work, or in the knowhow of expert mechanics, electricians, potters, and so on. Thus, most of an individual’s knowledge is just more or less shallow understanding or very limited practical knowhow, along with a more-or-less precise pointer to expert knowledge (Rabb et al., 2019). For instance, we know that “smoking causes lung cancer” but most of us are not sure why. So the neurobiological representations under study are really mostly pointers to knowledge that experts have or to pathways of transmission by which we can reliably access that information. Hence, the network that anchors much of our knowledge about the causal structure of the world is actually a network that sits across brains, not within a brain: It is not an aggregate of brain contents, but a pattern of interactions among brains with certain contents. Because it is the contents that are important, and not the specific brains, there are an unlimited number of patterns of interactions that would generate and maintain the same causal beliefs.

But the actual justification for those beliefs is more systematic than that. We have seen that it depends on community norms for attributing knowledge and associated institutions of knowledge certification. Within a given community, whatever complies with those norms qualifies as knowledge. Some communities may have rather eccentric norms, and regard some things as general knowledge that another community regards as wild-eyed conspiracy theory (issues of fake news and slander come to mind). Accordingly, an account of most of our knowledge will need to include the role of such social institutions and norms. I can legitimately claim to know that the sun does not revolve around the earth, that anthropocentric climate change is real, that the Pythagorean Theorem is true, and a great many other things I “learned in school,” even if I cannot myself produce proofs for any of them, or even say precisely what they amount to (Note that this is different from the case in which I could produce a proof if I sat down and tried to work one out). I know these things because they are known by recognized knowledge sources and I got them from socially recognized reliable transmitters of knowledge. This holds even if I can’t now remember where I learned it and am not capable of coming up with the evidence or proofs that sit in the heads of others.

My indirect and usually very superficial knowledge is anchored in the social network of experts and paths of transmission. Similarly, even the knowledge of experts is typically anchored in large part in that of other experts, as architects rely on results in materials science, industrial design, designers and manufacturers of drafting tables and instruments, and so on. Again, an enormous amount of anyone’s knowledge exists only by way of a larger community of cognizers and their interactions. These aspects of knowledge—including knowledge worked out in the privacy of my study or laboratory—are “knowledge” only by virtue of being anchored in a larger social network, independently of the particular neurobiology they are grounded in.

Consider a team of researchers writing a manuscript together. A complete account of collaboration and outsourcing involved in joint manuscript writing would have to include not only the brains of the authors, but also those whose evidence or testimony provides the support for claims made in the manuscript. If the manuscript presents findings summarizing a report, then the network would have to include the brains of everybody who wrote the report, or perhaps only those who contributed relevant parts. But how would you decide whose brain is relevant? It would depend on whether relevant knowledge was referenced in the manuscript. In other words, the structure of the knowledge is necessary to determine the relevant source and corresponding neural network to represent that knowledge. The knowledge would therefore not be reducible to a neural network, because identifying the network would depend on the knowledge.

Anyone attempting to describe the cross-brain neural network involved in writing a given manuscript, in the relevant processing and transmission (or lack thereof) of various sorts of information from multiple diverse sources, would not know which brains to look at, or what to look for in different brains, without already being able to identify how each bit of information in the manuscript is grounded. But even if we could identify *a posteriori* the network of brains or profiles of brain activity pertinent to a given piece of collaborative writing, we would be no further in explaining how or why the article came to be written. The reason that some ideas enter into a representation is because they elaborate on or integrate the representation in a more or less coherent way. One reason a report gets cited in a manuscript is that it supports or illustrates some informational point. If there is resonance among neural networks, it is because the information they represent is resonant; the neural networks are secondary. The knowledge held by the community is driving; any emergent neural networks are coming along for the ride.

At the beginning of this essay, we stated three widely-held assumptions in cognitive neuroscience that are inconsistent with facts about what and how people know. Our aim is not to diminish the important contributions of cognitive neuroscience. The assumptions we stated do hold for a variety of critical functions: Procedural knowledge is held in individual brains (or at least individual nervous systems in interaction with the world), and people obviously retain some symbolic knowledge in their individual brains. Moreover, common sense is enough to indicate that knowledge at a basic-level (Rosch, 1978) is regularly transferred between individuals. But far more symbolic knowledge than people are aware of is held by others – outside the individual’s brain. Thus, the purpose of much of cognitive neuroscience, to reduce knowledge to the neural level, is a pipe dream. The fact of communal knowledge creates a key limitation or boundary conditions for cognitive neuroscience.

## Summary and Implications

We have elaborated a theory of the community of knowledge, identifying as primary components outsourcing and collaboration, along with an hypothesis about how we construct epistemic pointers to potential sources of knowledge, whether those sources be people to whom we outsource knowledge or with whom we might collaborate. Our hypothesis places limits on the power of cognitive neuroscience to explain mental functioning (Text Box 1). Cognitive neuroscience has often focused on tasks that, at least on their face, are performed by individuals (cf., Becchio et al., 2010; Schilbach et al., 2013). But the limited predictive power of these tasks for human behavior may reflect the fact that these tasks and methods do not capture normal human thinking and may explain some of the limited replicability and generalization of fMRI findings (Turner et al., 2019). People devote themselves to tasks that involve artifacts and representational media designed by other people, to issues created by other people, to ideas developed by and with other people, to actions that involve other people, and of course to learning from sources outside themselves. None of these tasks are amenable to a full accounting from cognitive neuroscience.

**Box 1. Cognitive neuroscience meets the community of knowledge.**

Our understanding of how the world works is limited and we often rely on experts for knowledge and advice. One way that we rely on others is by *outsourcing* the cognitive work and task of reasoning to experts in our community. For example, we believe that “smoking causes lung cancer” even though many of us have little understanding of why this is the case. Here, we simply appeal to knowledge and expertise that scientists within our community hold.

And we behave in a manner that is consistent with knowing this information. We believe that smoking would elevate the risk of lung cancer; if a person were diagnosed with lung cancer, we would suppose they were a smoker; and we choose not to smoke because of the perceived cancer risk. But, again, an explanation for why “smoking causes lung cancer” is something that most of us do not know or understand. Our limited understanding simply relies on experts in the community who have this knowledge; we outsource the cognitive task of knowing and rely on experts for advice.

It may appear that this example is a special case and that we rarely outsource our knowledge to others. But, in fact, we do this all the time. Think of how well people understand principles of science, medicine, philosophy, history, and politics, or how modern technology works. We often have very little knowledge ourselves and instead rely on others to understand, think, reason, and decide. This reliance reflects how our individual beliefs are grounded in a *community of knowledge*.

By appealing to the community, we can ground our limited understanding in expert knowledge, scientific conventions, and normative social practices. Thus, the community justifies and gives meaning to our shallow knowledge and beliefs. Without relying on the community, our beliefs would become untethered from the social conventions and scientific evidence that are necessary to support them. It would become unclear, for example, whether “smoking causes lung cancer,” bringing into question the truth of our beliefs, the motivation for our actions, and no longer supporting the function that this knowledge serves in guiding our thought and behavior. Thus, to understand the role that knowledge serves in human intelligence, it is necessary to look beyond the individual and to study the community.

In this article, we explore the implications of outsourcing for the field of cognitive neuroscience: To what extent is cognitive neuroscience able to study the communal nature of knowledge? How would standard neuroscience methods, such as fMRI or EEG, capture knowledge that is distributed within the community?In the case of outsourcing, knowledge is not represented by the individual and knowledge is not transferred between individuals (i.e., it is the expert(s) who hold the knowledge). Thus, to study outsourcing, cognitive neuroscience would need to establish methods to identify the source of knowledge (i.e., who has the relevant information within the community?) and characterize the socially distributed nature of brain network function (e.g., what is the neural basis of outsourcing and the capacity to refer to knowledge held in the community?).

In this article, we identify the challenges this poses for cognitive neuroscience. One challenge is that representing the source of expertise for a given belief is not straightforward because expertise is time and context dependent, may rely on multiple members of the community, and may even depend on experts that are no longer alive. Another challenge is that outsourcing may reflect emergent knowledge that is distributed across the community rather than located within a given expert (e.g., knowledge of how to operate a navy ship is distributed across several critical roles; Hutchins, 1995). Standard methods in cognitive neuroscience, such as fMRI or EEG, are unable to directly assess knowledge distributed in the community because they are limited to examining the brains of individuals (or, at most, very small groups).

Thus, we argue that the outsourcing of knowledge to the community cannot be captured by methods in cognitive neuroscience that attempt to localize knowledge within the brain of an individual. We conclude that outsourcing is a central feature of human intelligence that appears to be beyond the reach of cognitive neuroscience.

Furthermore, our appeal to collective knowledge serves to reinforce the multiple realizability problem (Marr, 1982), allowing functional states to operate over complex and dynamic social networks. Whatever neural representations correspond to a bit of knowledge, they are tied to my belief by virtue of a functional relation (a placeholder in my brain that expresses the equivalent of “experts believe this!”), along with the existence of a reliable pedigree for that belief, not simply because my brain is part of a larger neural network. Functional states reflect communal knowledge. Because the human knowledge system is distributed across people, the parts of it that are anchored in others’ knowledge are beyond the reach of cognitive neuroscience.

In sum, the community of knowledge hypothesis implies that it’s a mistake to think of neurobiology as sitting beneath and potentially explaining the cognition that constitutes the emergent thinking in which groups and communities engage. And that’s most thinking. It also implies that components of that socially distributed cognitive system cannot in principle be defined in terms of or eliminated in favor of neurobiology.

Notice that our argument against reductionism has nothing to do with the nature of consciousness, the target of many such arguments (Searle, 2000; Dennett, 2018). In our view, this is a virtue because consciousness has escaped serious scientific analysis and therefore provides little ground for a serious scientific argument. The representations entailed by collective cognition, in contrast, can be analyzed. In principle, the representations involved in (say) designing a complex object may be abstract in the sense that they reflect interactions among knowledge stored in multiple brains, as well as the physical and virtual worlds, but they are describable nonetheless. As such, the emergent features of human cognition that we are advocating are well-documented and well-established as subjects of fruitful scientific research.

Our argument does have positive implications about how to make progress in cognitive neuroscience. To mention only some of the most basic of these, it suggests that our models of information processing for most tasks should focus on communal, not individual, representations. Because most of what we know and reason about is stored outside our heads, our models should not be exclusively about how we represent content, but also about how we represent pointers toward knowledge that is housed elsewhere. Because our actions are joint with others, models of information processing require not only a notion of intention, but a notion of shared intention (Tomasello et al., 2005). Finally, models of judgment that apply to objects of any complexity need to address how we outsource information, not just how we aggregate beliefs and evidence.

## Conclusion

The goal of this article is to focus cognitive neuroscientists on important facts about cognitive processing that have been neglected, and that, if attended to, would facilitate the project of cognitive neuroscience. Greater understanding of how people collaborate would help reveal how neural processing makes use of group dynamics and affiliation, and it would support a more realistic model of mental activity that acknowledges individual limitations. Greater understanding of how people outsource would help reveal the actual nature and limits of neural representation, and shed light on how people organize information by revealing how they believe it is distributed in the community and the world. And greater appreciation of the emergent nature of knowledge in society would help us recognize the limits of cognitive neuroscience, that the study of the brain alone cannot reveal the representations responsible for activities that involve the community. Thus, we join the call for a new era in cognitive neuroscience, one that seeks to establish explanatory theories of the human mind that recognize the communal nature of knowledge and the need to assess cognitive and neural representations at the level of the community – broadening the scope of research and theory in cognitive neuroscience by recognizing how much of what we think depends on other people.

## Data Availability Statement

The raw data supporting the conclusions of this article will be made available by the authors, without undue reservation.

## Author Contributions

All authors listed have made a substantial, direct and intellectual contribution to the work, and approved it for publication.

## Author Disclaimer

The views and conclusions contained herein are those of the author and should not be interpreted as necessarily representing the official policies or endorsements, either expressed or implied, of the ODNI, IARPA, DARPA, or the U.S. Government. The U.S. Government is authorized to reproduce and distribute reprints for Governmental purposes notwithstanding any copyright annotation thereon.

## Conflict of Interest

The authors declare that the research was conducted in the absence of any commercial or financial relationships that could be construed as a potential conflict of interest.

## Publisher’s Note

All claims expressed in this article are solely those of the authors and do not necessarily represent those of their affiliated organizations, or those of the publisher, the editors and the reviewers. Any product that may be evaluated in this article, or claim that may be made by its manufacturer, is not guaranteed or endorsed by the publisher.

## Footnotes

1 Geana, M., Rabb, N., and Sloman, S. A. (2021). *Walking the Party Line: The Growing Role of Political Ideology in Shaping Health Behavior in the United States.* Manuscript under review.
